# Compact Ultra Wide Band Microstrip Bandpass Filter Based on Multiple-Mode Resonator and Modified Complementary Split Ring Resonator

**DOI:** 10.1155/2013/402539

**Published:** 2013-11-04

**Authors:** J. Antonio Marcotegui, Jesús Miguel Illescas, Aritz Estevez, Francisco Falcone

**Affiliations:** ^1^R&D Department, Tafco Metawireless, Polıgono Plazaola, 31195 Aizoain, Spain; ^2^Electrical Engineering Departament, Universidad Publica de Navarra, Campus Arrosadıa, 31006 Pamplona, Spain

## Abstract

A new class of broadband microstrip filters for Ultra Wide Band (UWB) applications is proposed. In the design, different stages of parallel-coupled microstrip line and other stages with a Modified Complementary Split Ring Resonator (MCSRR)—a concept proposed here for the first time—are adjusted to obtain the desired response with broadband, sharp rejection, low insertion loss, and low return loss. Full wave simulation results as well as measurement results from fabricated prototypes are presented, showing good agreement. The proposed technique offers a new alternative to implement low-cost high-performance filter devices, applicable to a wide range of communication systems.

## 1. Introduction

 Since the unlicensed use of Ultra Wide Band (UWB) devices was authorized by the US Federal Communications Commission (FCC) in 2002, there has been a growing interest for the design of several components in applications like vehicular radar, wall imaging, indoor and hand-held UWB systems, among others [1]. In particular, for the design of wide bandpass filters different solutions have been proposed to solve the UWB and new wireless communications requirements [2, 3]. One of the main challenges in the use of this type of technology is the need to control undesired interference, due to its impact on overall system performance. Due to the large bandwidth required, the implementation of high-performance filters is necessary in order to guarantee successful operation.

Bandpass filters using multiple *λ*/4 parallel-coupled line resonators in planar technology have been studied and used in RF and microwave integrated circuits and systems for a long time [4]. A basic Multiple-Mode Resonator (MMR) based on a low-impedance stage and parallel-coupled line sections was proposed by Zhu et al. [5, 6]. In his work, the different resonant modes of the constituted MMR were used to achieve a wide passband microstrip filter, with five transmission poles, exhibiting high performance and compact size [6–8].

On the other hand, Split Rings Resonators (SRRs) were first introduced by Pendry [9] in order to produce artificial media with a strong magnetic response at microwaves and radiofrequencies. In his seminal work Pendry predicted that artificial media with negative permeability could be designed by using SRRs. Later, Smith's experiments [10] led to the first experimental realization of left handed media (LHM) predicted by Veselago [11]. There are interesting properties of SRRs that have been studied and demonstrated [12–14]. One of the main properties for our aim is that the first SRR resonance is quasistatic [12, 13], so that SRRs electrical size at such resonance is much smaller than the wavelength. Another particle, namely, the Complementary Split Ring Resonator (CSRR), which is the negative image of an SRR, has been recently proposed [15]. It has also been demonstrated that CSRRs etched on the ground plane or in the conductor strip provide a negative effective permittivity to the structure, which implies a stopband behavior near the first resonant frequency. In particular the small electrical size of the SRRs and CSRRs suggests the possibility of applying these particles for the design of compact microstrip or coplanar resonators and filters [16, 17].

 In this paper, a novel UWB bandpass filter combining MMR and CSRR with a capacitive gap techniques is proposed. Simulation as well as measurement results will be presented in Section 2, demonstrating the viability of this approach for the implementation of UWB compatible filters. 

## 2. Design of the Proposed UWB Filter

**Figure 1:**
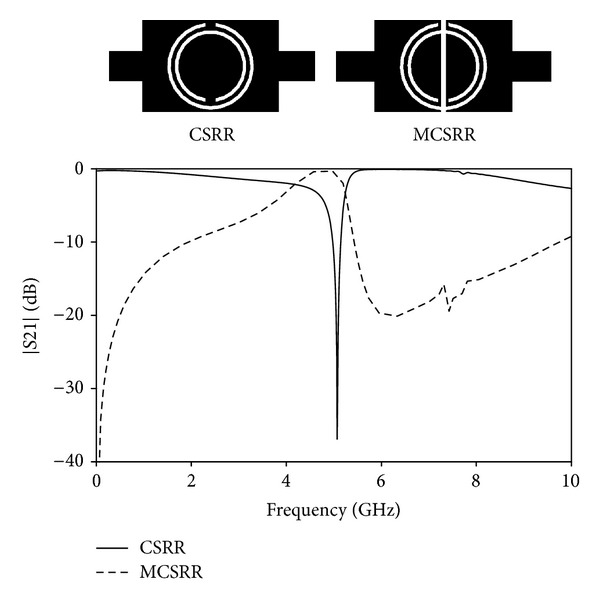
Layout of a CSRR and MCSRR etched in the strip conductor of a microstrip line and simulated S21 for CSRR (solid line) and MCSRR (dashed line).

It is well known that CSRRs can be excited by an electric field in the axial direction of the rings [15]. Due to the bianisotropy property of these particles they can also be excited in their first resonance by a transversal magnetic field perpendicular to the slits [12, 13]. If the CSRR is properly excited, it provides a negative effective permittivity as it has been explained in the previous section. Combining the CSRRs with a negative permeability media simultaneously negative permeability and permittivity that provides a bandpass could be obtained as it has been demonstrated [18]. The negative permeability media can be implemented by adding series capacitive gaps in the conductor strip. In this case the capacitive gap is placed in the middle of the CSRR which forms a pair of resonators coupled by the gap as it is depicted in Figure 1. The particle formed by a CSRR and the capacitive gap will be called Modified Complementary Split Ring Resonator (MCSRR) for simplicity. In Figure 1, it can be seen that the stopband response of the CSRR changes to passband when the capacitive gap is introduced. The resonant frequency of the CSRR that depends on the dimensions of the ring fits in with the passband of the MCSRR and that frequency can be calculated following [19], in which the dispersion relation can be derived by the following expression:

(1) $$\begin{array}{c} \cos\left(\beta l\right)=1+\frac{L\omega -\left(1/C_{g}\omega \right)}{2\left(L_{c}\omega /\left(1-L_{c}C_{c}\omega^{2}\;\right)\;-1/C\omega \right)}, \end{array}$$

where *C*_*g*_ is the CSRR gap inductance, *L*_*c*_ and *C*_*c*_ are the inductive and capacitive components of the equivalent *LC* tank, and *L* and *C* correspond to the inductive and capacitive behavior of the transmission line.

**Figure 2:**
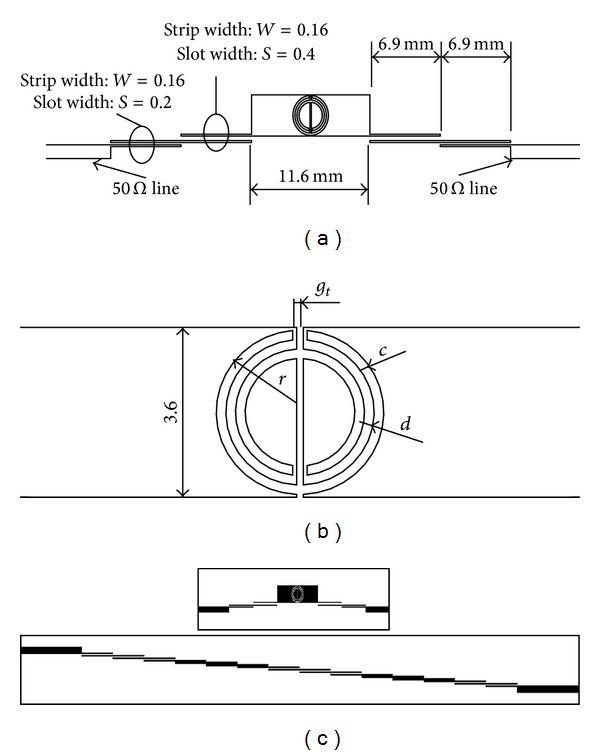
(a) Schematic of the compact UWB microstrip bandpass filter proposed. (b) Detail of the Modified Complementary Split Ring Resonator section. (c) Comparison between proposed filter and 14th-order Butterworth filter size with similar performance. Substrate: Rogers RO3010, *ε*_*r*_ = 10.2, thickness = 1.27. Unit: mm.

The schematic of the proposed compact UWB microstrip-line filter is depicted in Figure 2. It consists of a low-impedance strip with a MCSRR and two *λ*/4 parallel-coupled lines at each side. In this design of MMR, the first three resonant modes are taken into account together making up the passband as it is explained in [7]. In the presented filter the lower band is determined by the first resonant mode. The upper cut-off frequency is determined by tuning the MCSRR instead of the third resonant modes used for the cutoff frequency in [7]. Moreover by adding the MCSRR in the MMR design an additional control in the bandwidth is obtained, a fractional bandwidth that varies from 4% to 50% can be achieved and will be presented in future works, and the rejection level is also improved with respect previous solutions.

Another important feature in the design of this filter is that the ground plane has not been modified, which implies an important advantage in the integration of the device in solutions like Surface Mount Assembly (SMA) or Monolithic Microwave Integrated Circuits (MMICs).

## 3. Simulation and Experimental Results

**Figure 3:**
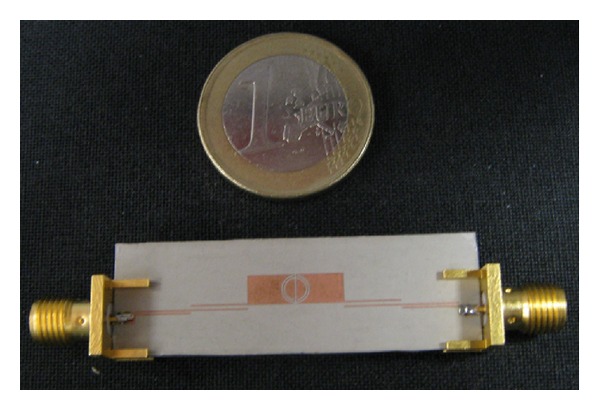
Fabricated prototype in Rogers RO3010 substrate, with 3.5 m D input/output connectors.

After an optimization procedure, the UWB prototype in Figure 2 was fabricated using a Rogers RO3010 substrate of thickness *h* = 1.27 mm, relative dielectric permittivity *ε*_*r*_ = 10.2, and metallization thickness 35 *μ*m by means of standard photo/mask etching technique. The CSRR, which has been designed following the dispersion relation previously presented in order to fix the frequency position of the quasistatic resonance, has an external radius *r* = 1.74 mm and strip width *c* = 0.2 mm with a separation between the inner and the outer ring *d* = 0.1 mm. The gap width that crosses the CSRR is *g*_*t*_ = 0.14 mm. The section of parallel-coupled lines near the central stage has a strip width *W* = 0.16 mm and a slot width of *S* = 0.4 mm. The sections of parallel-coupled lines near the port have strip width of *W* = 0.16 mm and a slot width of *S* = 0.2 mm. The prototype has been measured by using an *Agilent-8722* vector network analyzer. Full wave simulation results have been obtained with the aid of CST Microwave Studio Finite Integration Time Domain code, as well as in-house implemented FDTD code. To gain insight in the achieved size reduction, a schematic of an equivalent 14th-order Butterworth filter has also been designed and is depicted in the bottom portion of Figure 2. The fabricated prototype is shown in Figure 3, with 3.5 mm SMA input/output connectors for the measurement setup.

**Figure 4:**
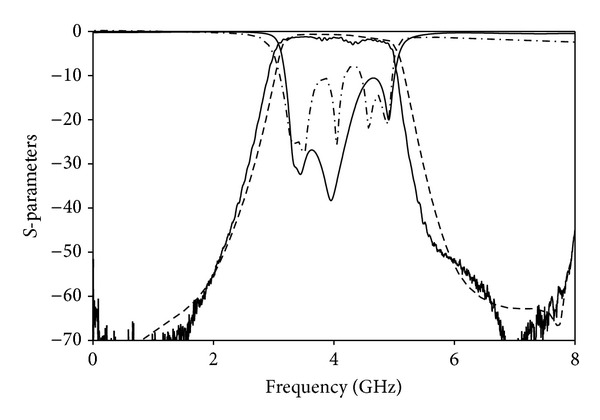
measured S21 (solid thick line), simulated S21 (dashed line), measured S11 (dash-dotted line), and simulated S11 (solid thin line).

The frequency response of the filter is depicted in Figure 4. Good agreement between simulation and measured results is observed, revealing a high performance for this filter. The passband is found to be between 3.1 and 4.9 GHz that corresponds to the first band in UWB systems. The measured insertion losses are lower than 2.5 dB in all passband which is mainly attributed to the ohmic conductor losses. Over the passband the return losses are found to be less than −10 dB, and the measured S11-curve demonstrates the five-pole resonator performance of the filter. One of the main features of this filter is the sharp-rejection in the stopband. More than 60 dB is achieved below 1.85 GHz in the lower band and from 6.4 to 7.8 GHz in the upper band which implies an improvement compared to previous solutions. The group delay is less than 2 ns in all passbands and the maximum variation is about 0.7 ns which demonstrates the good linearity of the device.

**Figure 5:**
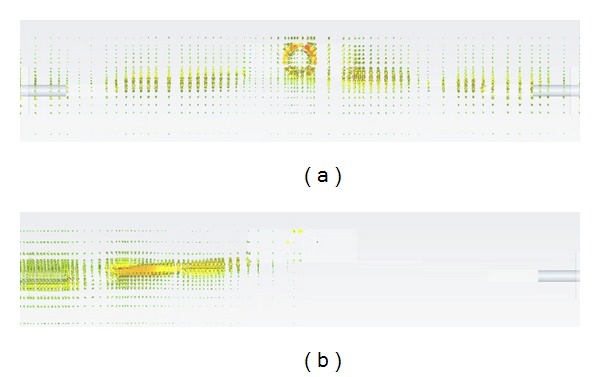
Surface current density plots obtained by full wave simulation: (a) operation in the passband region of the filter, at 3.6 GHz; (b) operation in the stopband region of the filter, at 6 GHz.

In order to gain insight in the operation of the proposed filter, simulation results for the surface current density at two different frequencies of operation, 3.6 GHz (in the central portion of the passband) and 6 GHz (in the stopband region), are depicted in Figure 5. As it can be seen, in the case of operation within the passband, surface currents are present from input to output of the device, which is straightforward from the fact that low losses are present. It is interesting to notice that there is a strong concentration of surface currents within the MCSRR element (in the central part of the figure), which is due to the fact that the quasistatic resonance is being fully excited. On the other hand, the lower image in Figure 5 shows the surface current density at 6 GHz, which is in the stopband region of operation of the device. In this case, reflection can be seen, which is given by the fact that the MCSRR element is not being excited and, therefore, provides a strong rejection in an otherwise passband structure. Therefore, the combination of the frequency response of the host coupled line structure with the MCSRR element gives rise to the desired frequency response in the FCC UWB spectrum.

Depending on the given specifications, the filter size could be compacted by reducing the number of parallel-coupled lines stages. The bandwidth can be also adjusted, depending on the specifications of the filter optimizing the circuit dimensions. If this filter is compared with a classical Butterworth filter, implemented with parallel-coupled lines with similar performance, that is, fractional bandwidth FBW = 50%, centered at *f*_*o*_ = 4 GHz and −60 dB in the stopband, it would be necessary a 14th-order filter (see Figure 2), that is, fourteen *λ*/4 parallel-coupled line stages (96.6 mm of total length). Moreover in the conventional filter design a spurious band is present at 2*f*_*o*_ while the filter presented in this paper exhibits near 60 dB rejection at that frequency.

## 4. Conclusions

A new UWB filter using parallel-coupled lines and Modified Complementary Split Ring Resonators has been presented, as well as the basis for its design. The main characteristic of this design is the inherent flexibility in order to obtain a specified filter response. In this paper a filter for the first UWB range with high performance is proposed. Bandwidth and rejection levels are controlled by including the MCSRR in a MMR. The proposed design achieves high performance (insertion losses lower than −2.5 dB, return losses lower than −10 dB and sharp-rejection values, higher than 60 dB, near the passband) in a compact lay-out configuration. In order to produce designs more robust against interferences and radiation losses, stripline technology will be employed in future works. The proposed design offers high performance and simple fabrication for the implementation of UWB filters, which can be modified to be suitable for other types of communication systems.
